# Cesarean-section Rates in Brazil from 2014 to 2016: Cross-sectional Analysis Using the Robson Classification

**DOI:** 10.1055/s-0040-1712134

**Published:** 2020-06-19

**Authors:** Roxana Knobel, Thiago Jose Pinheiro Lopes, Mariane de Oliveira Menezes, Carla Betina Andreucci, Juliana Toledo Gieburowski, Maira Libertad Soligo Takemoto

**Affiliations:** 1 Department of Gynecology and Obstetrics, Universidade Federal de Santa Catarina, Florianópolis, Santa Catarina, Brazil; 2 Postgraduate Program in Tocogynecology, Faculty of Medicine, Universidade Estadual Paulista, Botucatu, SP, Brazil; 3 Department of Medicine, Universidade Federal de São Carlos, São Carlos, SP, Brazil

**Keywords:** cesarean section, vaginal birth after cesarean section, obstetric delivery, repeat cesarean section, induced labor, cesárea, nascimento vaginal após cesárea, parto obstétrico, cesárea repetida, trabalho de parto induzido

## Abstract

**Objective** To obtain cesarean-section (CS) rates according to the Robson Group Classification in five different regions of Brazil.

**Methods** A descriptive epidemiological study using data from secondary birth records from the Computer Science Department of the Brazilian Unified Health System (Datasus, in Portuguese) between January 1st, 2014, and December 31st, 2016, including all live births in Brazil.

**Results** The overall rate of CS was of 56%. The sample was divided into 11 groups, and vaginal births were more frequent in groups 1 (53.6%), 3 (80.0%) and 4 (55.1%). The highest CS rates were found in groups 5 (85.7%), 6 (89.5%), 7 (85.2%) and 9 (97.0%). The overall CS rate per region varied from 46.2% in the North to 62.1% in the Midwest. Group 5 was the largest obstetric population in the South, Southeast and Midwest, and group 3 was the largest in the North and Northeast. Group 5 contributed the most to the overall CS rate, accounting for 30.8% of CSs.

**Conclusion** Over half of the births in Brazil were cesarean sections. The Midwest had the highest CS rates, while the North had the lowest. The largest obstetric population in the North and in the Northeast was composed of women in group 3, while in the South, Southeast and Midwest it was group 5. Among all regions, the largest contribution to the overall CS rate was from group 5.

## Resumo

**Objetivo** Identificar as taxas de cesárea de acordo com a Classificação de Robson nas cinco regiões do Brasil.

**Métodos** Estudo epidemiológico descritivo utilizando dados secundários obtidos do Departamento de Informática do Sistema Único de Saúde (Datasus) entre 1° de janeiro de 2014 e 31 de dezembro de 2016, incluindo todos os nascidos vivos no Brasil.

**Resultados** Cesáreas representaram 56% de todos os nascimentos. A amostra foi dividida em 11 grupos, e partos vaginais foram mais frequentes nos grupos 1 (53,6%), 3 (80,0%) e 4 (55,1%). As maiores taxas de cesárea foram encontradas nos grupos 5 (85,7%), 6 (89,5%), 7 (85,2%) e 9 (97,0%). A taxa geral de cesárea variou de 46,2% no Norte a 62,1% no Centro-Oeste. O grupo 5 representou a maior população obstétrica no Sul, Sudeste e Centro-Oeste, e o grupo 3, no Norte e Nordeste. O grupo 5 contribuiu mais para a taxa geral de cesárea, totalizando 30,8%.

**Conclusão** Mais da metade dos nascimentos no Brasil ocorreu por cesárea. O Centro-Oeste apresentou a maior taxa, e o Norte, a mais baixa. A maior população obstétrica no Norte e no Nordeste foi o grupo 3, enquanto no Sul, Sudeste e Centro-Oeste foi o grupo 5. Entre todas as regiões, a maior contribuição para a taxa geral de cesárea foi do grupo 5.

## Introduction

Cesarean section (CS) is a surgical procedure that reduces maternal and neonatal morbidity and mortality when performed for clinical reasons. However, there is evidence that CS rates higher than 10% to 15% are associated with higher morbidity and mortality risks for the mother and the newborn. 1 2 Based on a global study of maternal and fetal complications in 24 countries, the World Health Organization (WHO) stated that CS is associated with higher risks than vaginal delivery, and should therefore be offered when a clear benefit is expected, offsetting the higher costs and additional risks. 3 Other concerns and controversies around mode of delivery include inequalities in the performance of CS not only among different countries, but also between the public and private systems within the same country, 4 and the costs imposed upon the already financially overburdened healthcare systems. 5

Recently, CS percentages regarding the total amount of births have increased worldwide, especially in middle and high-income countries, the latter especially affected by the obstetric transition phenomenon. 2 Brazil stands out with world's second highest CS rate, surmounted only by the Dominican Republic, 6 and over half of the births in the country are through CS. 4 The reasons CS rates are increasing are not simple to understand once they might combine financial, social, healthcare system, medical and cultural factors. 1

One way to tackle the issue of optimizing cesarean section practices is to identify whether there are specific groups of pregnant women contributing to the rise in the overall surgery rate, and subsequently to direct tailored interventions targeting their specificities. A 2011 WHO systematic review suggested that the Robson classification is the most appropriate system available to monitor and compare CS rates within a women-based classification. 7 The Robson classification is a prospective instrument based on six obstetric parameters (parity, previous CS, gestational age, onset of labor, fetal presentation, and the number of fetuses) that divides pregnant women into 10 groups. 8 9 Those groups are fully inclusive and mutually exclusive, meaning that every pregnant woman will fit into one of them and no more than one. Its simplicity, reproducibility and clinical relevance have led to its universal adoption in recent years, with endorsement from the WHO. 9 10

In 2014, the Brazilian Ministry of Health chose to apply the Robson classification to its annual live birth statistics, since then enabling the national assessment of the association of selected obstetric parameters with mode of delivery. In this context, the present study aims to address CS rates according to the Robson classification in the five geographic regions of Brazil, to provide evidence to better understand and outline strategies to help reduce the high CS rate in the country.

## Methods

The present is a descriptive epidemiological cross-sectional study using secondary database data of the Computer Science Department of the Brazilian Unified Health System (Datasus, in Portuguese) from 2014 to 2016. The study population includes all live births in Brazilian territory within the selected period. Geographically, the Brazilian territory is divided into five regions: North, Northeast, Midwest, Southeast and South, and the stratification was included in the analyses. When information on mode of delivery was not available, the subjects were excluded ( *n* = 10,503; < 0.01% of total births).

Data was obtained from the Ministry of Health's Live Birth Information System (Sinasc, in Portuguese), through the Datasus online platform, using the Tabnet application, which was developed by Datasus. 10 All live births that occur in the country receive a unique record in the Sinasc database, which comprises mandatory birth notification data as defined by the Brazilian federal government and includes all births: vaginal, instrumental and CS births both from public and private institutions, as well as out-of-hospital births (including planned and unplanned homebirths). This database also includes information about birth dates, time and location, as well as maternal and newborn characteristics. The Sinasc is an effective tool to assess information on births in Brazil, covering more than 90% of all births nationwide. 11 In the present study, data were extracted filtered by region, using the dependent variable “Robson's groups” and the independent variable “mode of delivery.”

The Robson Classification comprises a categorization of pregnant women into ten groups at the time of their admission for birth. 8 The classification is based on six obstetric characteristics shown in Table 1 .

**Table 1:** Overall cesarean section (CS) rate and in each Robson group in Brazil

Robson classification	Cesarean section rates (%)
1. Nulliparous, single cephalic, ≥ 37 weeks, in spontaneous labor	46.4
2. Nulliparous, single cephalic, ≥ 37 weeks, induced or CS before labor	69.0
3. Multiparous (excluding previous CS), single cephalic, ≥ 37 weeks, in spontaneous labor	20.0
4. Multiparous (excluding previous CS), single cephalic, ≥ 37 weeks, induced or CS before labor	44.9
5. Previous CS, single cephalic, ≥ 37 weeks	85.7
6. All nulliparous breeches	89.5
7. All multiparous breeches (including previous CS)	85.2
8. All multiple pregnancies (including previous CS)	82.8
9. All women with a single pregnancy in transverse or oblique lie (including those with previous CS)	97.0
10. All single cephalic, < 37 weeks (including previous CS)	50.3
11. Births not classified in any groups due to lack of information	59.1
Total	56.0

Births not classified in any groups due to lack of information were included in the present study under the unofficial terminology “group 11.”

The outcomes in the present study included national and regional data on: a) CS rates according to each Robson group; b) obstetric population size in each Robson group; and c) the relative contribution of each Robson group to the overall CS rate in Brazil.

## Results

Cesarean section was the most common mode of delivery in the country in the 2014–2016 period, comprehending 56% of all births ( Table 1 ). Only three Robson groups had a higher proportion of vaginal deliveries when compared with the proportion of CSs: groups 1, 3, and 4. The highest CS rates were found in the multiparous group with a history of previous CS and single cephalic fetus at term (group 5), in non-cephalic presentations in general (groups 6, 7 and 9) and in multiple pregnancies (group 8), as shown in Table 1 .

The overall CS rate ranged from 46.2% in the North to 62.1% in the Midwest ( Table 2 ). The Midwest showed the highest CS rates in the 5 largest groups of pregnant women (groups 1 to 5), while the lowest CS rates had a heterogeneous distribution between the regions.

**Table 2:** Cesarean section rate (%) in each Robson group by region

Robson group	North	Northeast	Midwest	Southeast	South
1	42.5 ^†^	45.8	53.8*	46.9	45.2
2	68.3	63.6 ^†^	73.4*	69.1	72.5
3	17.4	21.9	24.0*	18.9	16.9 ^†^
4	46.9	45.3	50.4*	43.0 ^†^	46.7
5	80.5 ^†^	85.6	87.5*	86.4	85.7
6	89.6	83.9 ^†^	90.5	91.1	93.2*
7	86.0	79.2 ^†^	87.6	86.9	89.3*
8	78.3	75.4 ^†^	87.2	85.8	86.3*
9	98.0*	97.1	97.8	96.4 ^†^	96.9
10	38.5 ^†^	43.3	53.3	56.2	57.3*
11	50.9	50.5 ^†^	76.7	66.9	71.8*
Total	46.2	50.2	62.1*	59.7	61.2

Notes: *Highest values for each Robson group; ^†^ lowest values for each Robson group.

**Fig. 1:**
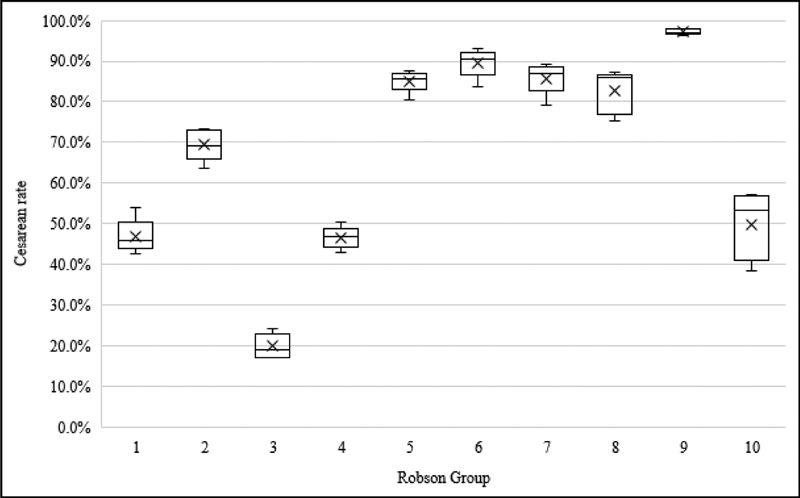
Interregional variability in the CS rate in each Robson group.

Fig. 1 presents a boxplot showing the variability in CS rates across the regions of Brazil for each Robson group. The “x” marker inside each box denotes the mean rate of CSs among the five regions, while the middle horizontal line represents the median rate. There are also whiskers above and below the boxes representing the maximum and minimum CS rates found for each group when comparing the 5 regions. Women with a single pregnancy in transverse or oblique lie – including those with previous CS (group 9) – had the smallest variability: only 1.6% among the 5 regions. The largest differences in CS rates among regions were identified in preterm cephalic births (group 10): from 38.5% in the North to 57.3% in the South (18.8% of absolute difference), and in multiple pregnancies (group 8): from 75.4% in the Northeast to 86.3% in the South (10.9% of absolute difference; Table 2 ).

The size of the Robson groups varied from region to region ( Table 3 ). Group 5 (all multiparous women with at least 1 previous CS with a single fetus, cephalic, ≥ 37 weeks) comprised the largest obstetric population in the South, Southeast, and Midwest, while group 3 (multiparous women without previous CS with a single fetus, cephalic, ≥ 37 weeks, in spontaneous labor) was the largest obstetric population in the North and Northeast.

**Table 3:** Relative distribution of live births (%) per Robson group

Robson group	North	Northeast	Midwest	Southeast	South	Brazil
1	21.3	22.0	18.3	14.3	13.4	17.4
2	6.7	10.4	12.6	20.3	21.7	15.6
3	27.2	23.5	17.5	13.6	13.2	18.1
4	5.4	7.3	7.7	11.9	12.0	9.6
5	16.3	16.2	22.7	22.4	22.8	20.1
6	1.1	1.3	1.6	1.5	1.8	1.4
7	1.8	1.7	2.4	1.7	2.1	1.9
8	1.5	1.8	2.0	2.3	2.2	2.0
9	0.3	0.3	0.2	0.2	0.3	0.2
10	9.9	9.4	8.2	8.8	8.5	9.0
11	8.5	6.2	6.7	3.1	1.9	4.6

The size of group 2 (single cephalic nulliparous women, ≥ 37 weeks, whose delivery was induced or who underwent CS before the onset of labor) varied significantly among regions, representing only 6.7% of all pregnant women in the North region, and 21.7% of all pregnant women in the South region. An opposite trend was observed in group 3 (multiparous women without previous CS, single fetus, cephalic, ≥ 37 weeks, in spontaneous labor): the lowest proportion was found in the South region (13.2%), and the largest, in the North region (27.2%).

In all regions, the group that most contributed to the overall CS rate was group 5, which accounted for 30.8% of CSs in the country ( Table 4 ). The second largest contribution to CS rates in the North, Northeast and Midwest was from group 1 (nulliparous, single fetus, cephalic, ≥ 37 weeks, in spontaneous labor), while in the South and Southeast, group 2 contributed the most for CS rates.

**Table 4:** Relative contribution of each Robson group (%) to the overall cesarean section rate by region

Robson group	North	Northeast	Midwest	Southeast	South	Brazil
1	19.6	20.1	15.9	11.2	9.9	14.4
2	10.0	13.2	14.9	23.4	25.7	19.2
3	10.3	10.2	6.8	4.3	3.7	6.4
4	5.5	6.6	6.2	8.5	9.2	7.7
5	28.5	27.5	32.0	32.5	31.9	30.8
6	2.1	2.1	2.4	2.2	2.8	2.3
7	3.4	2.7	3.4	2.5	3.0	2.8
8	2.5	2.7	2.9	3.3	3.1	3.0
9	0.7	0.5	0.3	0.3	0.5	0.4
10	8.2	8.1	7.0	8.2	8.0	8.1
11	9.4	6.2	8.2	3.4	2.2	4.9

## Discussion

More than 8 million births in Brazil from 2014 to 2016 were analyzed using the Robson classification system. It was possible to observe that CS is the most common mode of delivery both overall in the country and in all geographic regions, except for the North.

Cesarean section rates in Brazil were estimated in 30% in the early 1980s, reached 40% in the early 1990s, and exceeded 50% in 2012. 12 The dramatic increase in CS rates has multifactorial causes – that are not the main scope of the present article – and some possible reasons for the CS rates in the country to stand out are a common cultural belief that vaginal delivery is an uncontrollably painful process, fueled by infrequent adoption of non-pharmacological pain relief methods and low availability of regional anesthesia at Brazilian maternity facilities. At the same time, the media has historically pictured vaginal birth as a dangerous and unpredictable event, reinforcing the belief that adverse perinatal outcomes are direct consequences of the non-use of CS or delay in performing the surgery. 12 13 14 15 Another point that may contribute to CS rates is that in Brazil most births are performed by medical doctors. The role of midwives and nurse-midwives in childbirth assistance is limited and uneven in different locations in the country. 14

The rates of CS in each Robson group can vary in countries depending on the characteristics of the obstetric population. Therefore, there are no ideal rates established. The WHO Multi-Country study 16 applied data from selected health facilities with low CS and positive maternal and neonatal childbirth outcomes in 29 countries to create a global reference for CS rates. The findings indicated which CS rate could be achieved in each Robson group without worsening the obstetrical outcomes. Thus, groups 5 to 8 had the highest CS rates (74.4%, 78.5%, 73.8%, and 57.7% respectively), and groups 1 and 3 had the lowest (9.8% and 1.3% respectively). Group 2 had almost 40% of CSs, and group 4, a rate of 23.7%. The overall CS rate was 18.5%. 16

In the present study with Brazilian data, higher CS rates were found in the South, Southeast and Midwest regions. It is worth mentioning that private health system utilization is also higher in these three regions, 13 probably contributing to those rates, since CSs are more commonly performed at private health facilities in Brazil. 12 13 14 15 17 Higher education, better socioeconomic status and living in urban areas may also play a part in raising CS rates in the aforementioned regions, when compared with the North and Northeast. All of those aspects have already been historically associated with higher chances of CS. 18 The lower CS rates in the Brazilian Northern region may also be explained by sociocultural aspects or local obstetrical care characteristics, both of which were not addressed in the present study.

Higher overall CS rates were also found in geographic regions with the highest proportion of multiparous women who had previous CS and a single-term cephalic fetus (Robson group 5). The frequency of primary CS in nulliparous women in the recent past resulted in this group's expansion as one of its direct consequences. In this study, the highest CS rates in nulliparous, single-term, cephalic, term fetuses (Robson groups 1 and 2) were found in the Midwest region, which was also the location with the highest overall CS rate (62.1%).

The largest obstetric population in Brazil was classified as Robson group 5, which had the largest participation in the overall CS rate in all 5 geographic regions as well (almost 1/3 of all surgeries). Therefore, a substantial impact over the country's overall CS rate could be achieved in the future by applying specific interventions addressing directly this group of women with previous uterine scars. For instance, the decrease in CS rates in nulliparous women could lead to a decline in the population size of group 5, and to an increase in Groups 3 and 4, in which CS rates are 3 times lower. Additionally, a trial of labor should be offered to multiparous women with previous CS who choose to have vaginal delivery, as stated by the Royal College of Obstetricians and Gynecologists, 19 the American College of Obstetricians and Gynecologists, 20 and the Brazilian guidelines, 4 which could result in a direct decrease in CS rates in Group 5.

Robson groups 1 and 2 (nulliparous single-term cephalic fetuses) accounted for approximately 1/3 of all CSs in every region of the country. While in Brazil groups 1 and 2 combined represent a CS rate of 57.1%, in France they reach 23.2%, 21 and, in Sweden, 14.3%. 22 Brennan et al 23 analyzed nine institutional cohorts from nine countries and found that CS rates in these two groups can largely explain the variations in the overall CS rate in different settings. Therefore, efforts to reduce the overall CS rate should also focus on managing these groups of nulliparous women.

Non-cephalic presentations (groups 6, 7 and 9), multiple pregnancies (group 8) and preterm births (group 10) displayed a very similar proportion within the obstetric population in each region, and had a relatively small contribution to the overall CS rate due to their reduced absolute magnitude. The external cephalic version technique in non-cephalic presentations could decrease the population size of these groups in which CS rates are very high, contributing to reduce the overall CS rate.

The births recorded as Group 11 (not classified in Robson's group due to lack of required parameters) were scarce, especially considering that the Ministry of Health only recently adopted the classification. The highest underreporting rate was found in the North (8.5%), and the lowest, in the South (1.9%).

Considering the current evidence advocated by the WHO that CS rates higher than 10% are not associated with a reduction in maternal and neonatal mortality rates, 24 25 the use of the Robson classification comparing CS rates and obstetrical outcomes is a way to contribute to future discussions on the topic. 10 In the present study, applying the Robson classification enabled us to identify specific obstetric characteristics of women who underwent CS. Compared with having one single national or regional CS rate, to understand the factors associated with having a CS under the perspective of the Robson groups might enable a much broader analysis of the Brazilian context. The findings might therefore be employed to design health policies addressing those specific population groups in the future and tackle the issue of the increasing CS rates in the country.

The present study has several limitations. First, it is a secondary analysis based on the Sinasc database, which prevented us from obtaining further details on clinical features available from hospital charts. Second, the data were extracted from a short period of time (from 2014 to 2016). Finally, Robson groups 2, 4 and 5 comprehend both women under labor induction or who underwent CS before the onset of labor. Since the two categories are not individualized, it is not possible to establish the role of labor induction upon birth outcome, and the weight of elective CS before the onset of labor might play a role on the global CS rates. Among the latter, 50% were scheduled CSs that, therefore, could not have been studied regarding possible associations to the global CS rates.

In the present study, it was possible to profile the CS rate in Brazil applying the Robson classification (ten-group classification) instead of using an absolute generic percentage to evaluate the heterogeneous obstetric population. The study sample was large, comprising 8,854,727 live newborns and few missing data on mode of delivery (< 0.01%).

Future studies comprising a larger time span might help understand the temporal trend of CS rates in Brazil. As previously published studies have already proposed, in the future, groups 2, 4 and 5 should be divided into subgroups: “a) labor induction; and b) cesarean section before the onset of labor.” 22 26 This would enable a proper evaluation of the burden of each of the conditions upon CS rates. The availability of data regarding maternal and perinatal outcomes through the Brazilian Ministry of Health together with information about mode of delivery and Robson Group classification would provide better means to analyze obstetrical practices in the country. The obtained data could contribute to the development of better care strategies and policies for the health of women and newborns. 27

## Conclusion

Most of births in Brazil occurred through CS. The Midwest region had the highest CS rate, while the North region had the lowest CS rate. The largest obstetric populations in the North and in the Northeast regions were included in group 3. In the South, Southeast and Midwest, the more prevalent population was included in group 5. Among all regions, the largest contribution to the overall CS rate was from group 5, accounting for 30.8% of CSs in the country.
